# Accurate scoliosis vertebral landmark localization on X-ray images via shape-constrained multi-stage cascaded CNNs

**DOI:** 10.1016/j.fmre.2022.10.014

**Published:** 2022-11-10

**Authors:** Zhiwei Wang, Jinxin Lv, Yunqiao Yang, Yi Lin, Qiang Li, Xin Li, Xin Yang

**Affiliations:** a Britton Chance Center for Biomedical Photonics, Wuhan National Laboratory for Optoelectronics, Huazhong University of Science and Technology, Wuhan 430074, China; b MoE Key Laboratory for Biomedical Photonics, Collaborative Innovation Center for Biomedical Engineering, Huazhong University of Science and Technology, Wuhan 430074, China; c Department of Computer Science, City University of Hong Kong, Hong Kong 999077, China; d Department of Computer Science and Engineering, The Hong Kong University of Science and Technology, Hong Kong 999077, China; e Department of Radiology, Union Hospital, Tongji Medical College, Huazhong University of Science and Technology, Wuhan 430022, China; f School of Electronic Information and Communications, Huazhong University of Science and Technology, Wuhan 430074, China

**Keywords:** Vertebra, X-ray, Landmark localization, Multi-stage, Cascade, Shape constraint

## Abstract

Vertebral landmark localization is a crucial step in various spine-related clinical applications, which requires detecting the corner points of 17 vertebrae. However, the neighboring landmarks often disturb each other because of the homogeneous appearance of vertebrae, making vertebral landmark localization extremely difficult. In this paper, we propose a multi-stage cascaded convolutional neural network (CNN) to split a single task into two sequential steps: center point localization to roughly locate 17 center points of vertebrae, and corner point localization to determine four corner points for each vertebra without any disturbance. The landmarks in each step were located gradually from a set of initialized points by regressing offsets using cascaded CNNs. To resist the mutual attraction of the vertebrae, principal component analysis was employed to preserve the shape constraint in offset regression. We evaluated our method on the AASCE dataset, comprising 609 tight spinal anteroposterior X-ray images, and each image contained 17 vertebrae composed of the thoracic and lumbar spine for spinal shape characterization. The experimental results demonstrated the superior performance of vertebral landmark localization over other state-of-the-art methods, with the relative error decreasing from 3.2e−3 to 7.2e−4.

## Graphical abstract

**Figure:**
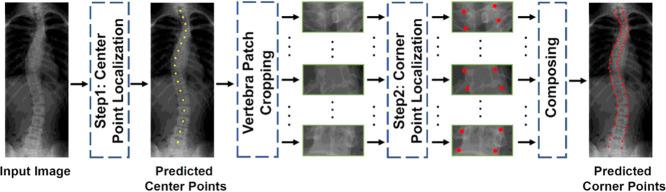

## 1 Introduction

Vertebral landmark localization is a key step in computer-aided diagnosis of spinal diseases, such as Cobb angle calculation, biomechanical load analysis, and detecting vertebral fractures and other pathologies [1]. However, manually identifying 17 vertebrae and localizing the four corner points of each vertebra is extremely time-consuming and infeasible for large-scale screening. Thus, automatic methods that detect vertebral landmarks, that is, 17×4=68 points in total, are in high demand. Although automatic vertebral landmark localization has been studied for decades, it remains challenging owing to high ambiguity and variability of X-rays images [2], superimposing heterogeneous soft tissue, and similar texture features of nearby vertebrae [1].

Two major families of approaches exist to address this challenging task: the heatmap-based and regression-based approaches.

**The heatmap-based approach** is one of the most successful approaches to vertebral landmark localization. It typically utilizes a convolutional neural network (CNN) to generate a heatmap with the same size of the input posterior-anterior X-ray image. The vertebral landmarks are subsequently obtained by finding points with a local maximum response in the heatmap. Zhang et al. [3] utilized four different fully connected layers to predict four heatmaps, each of which indicates a group of corner points with different semantics, that is, the top left, top right, bottom left, and bottom right points. The landmark coordinates are obtained indirectly by post-processing the predicted heatmap, i.e., finding local maximums’ column and row indices as the landmark coordinates. These indices are discrete integers, leading to non-trivial quantization errors in the continuous numerical ground-truth [4]. To address this problem, Yi et al. [5] proposed to first locate 17 center points of the whole spine based on heatmaps, and subsequently regress the numerical coordinates of the corner points relative to the center points using shape regression.

Despite the success of these heatmap-based methods, a common flaw can be often observed from their results, i.e., scattering false positives incurred by other vertebra-like structures and missing landmarks induced by the dilution of superimposed tissues. These incorrect predictions lead to the derivation of wrong clinical parameters, resulting in misdiagnosis. However, heatmap-based landmark localization approaches have been demonstrated to be robust in tasks with large pose variations, such as human pose estimation. Nonetheless, they might be overkilled in our task because human spines roughly fit a similar curve, that is, the pose variation is secondarily important.

For landmark localization, several studies have resorted to the transformer technique, which was originally proposed in the field of natural language processing (NLP) and has recently flourished in a variety of computer vision fields, including landmark localization. Most, if not all, transformer-based methods typically follow the basic pipeline of the heatmap-based approach and generate heatmaps to obtain landmark coordinates. Yang et al. [6] incorporated a transformer into a CNN to implicitly capture long-range spatial relationships of human body parts efficiently and then decoded these relationships into heatmaps for human pose estimation. Zhao et al. [7] focused on 3D landmark localization and combined the strengths of the graph convolution network (GCN) and transformer to characterize a set of pre-localized 2D landmarks and map them into their 3D counterparts. However, these transformer-based methods involve a self-attention mechanism and must compute similarity scores of all pairs of landmarks, which is prohibitively expensive, meanwhile requiring a large amount of data for training. To address this, Tao and Zheng [8] proposed a lightweight transformer for vertebral detection in 3D CT volumes; however, the vertebral landmark localization in 2D X-ray images is more challenging than that in 3D CT volumes owing to the superimposing tissues and less spatial information. The effectiveness of the transformer on vertebral landmark localization has not been studied and verified.

**The regression-based approach** is another approach to vertebral landmark localization, which utilizes an end-to-end CNN model to directly regress the coordinates. Sun et al. [2] proposed a structured support vector regression ($S^{2}\text{VR}$), which encourages multiple outputs to simultaneously share similar sparsity patterns such as spatial correlations. Wu et al. [9] constructed a relation matrix for spinal landmarks to explicitly enforce dependencies between the outputs. Although these methods can ensure one-to-one matches between the regressed points and ground truth points, directly regressing coordinates has two major problems: (1) CNNs may not be sufficiently powerful to precisely regress all landmarks in a single forward propagation, and (2) the points of each vertebra tend to be strongly attracted by other vertebrae during regression because they all share similar appearances. The affinity among vertebrae can cause a sudden point shift to their neighbors, which can barely be corrected if the shifted points are too far from their ground truth.

**Fig. 1:**
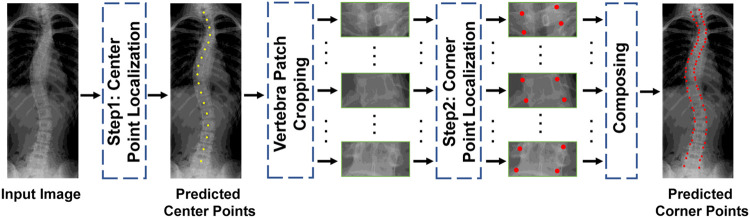
**Overview of the proposed two-step vertebral landmark localization methods.** See Figs. 2 and 3 for details of the two steps.

In this paper, we propose multi-stage cascaded CNNs for vertebral landmark localization, comprising two sequential steps : *center point localization* for vertebrae and *corner point localization* for each localized vertebra, as shown in Fig. 1. In the first step, we train three-stage cascaded CNNs to gradually update 17 center points of a vertebrae from 17 initial points. The output points of each former stage CNN are used as input to the subsequent stage. In each center-point localization stage, local features around each vertebra are first extracted separately and then concatenated to jointly regress all the center points of the vertebrae. After the first step, each vertebra is roughly localized to guide the following corner point localization step, which focuses on its own area, overcoming the point shifting problem caused by the affinity among the vertebrae. In the second step, another three-stage cascaded CNN is trained to gradually localize four corner points for each vertebra, and output the final 68 landmarks. To further address the mutual attraction problem among the vertebrae during regression, we propose regressing the PCA-transformed coordinate offsets instead of the direct absolute offsets using a CNN in each stage. Thus, the location of a single point can be constrained using a global shape constraint, that is, a predefined variance of distance between two adjacent vertebrae.

**Fig. 2:**
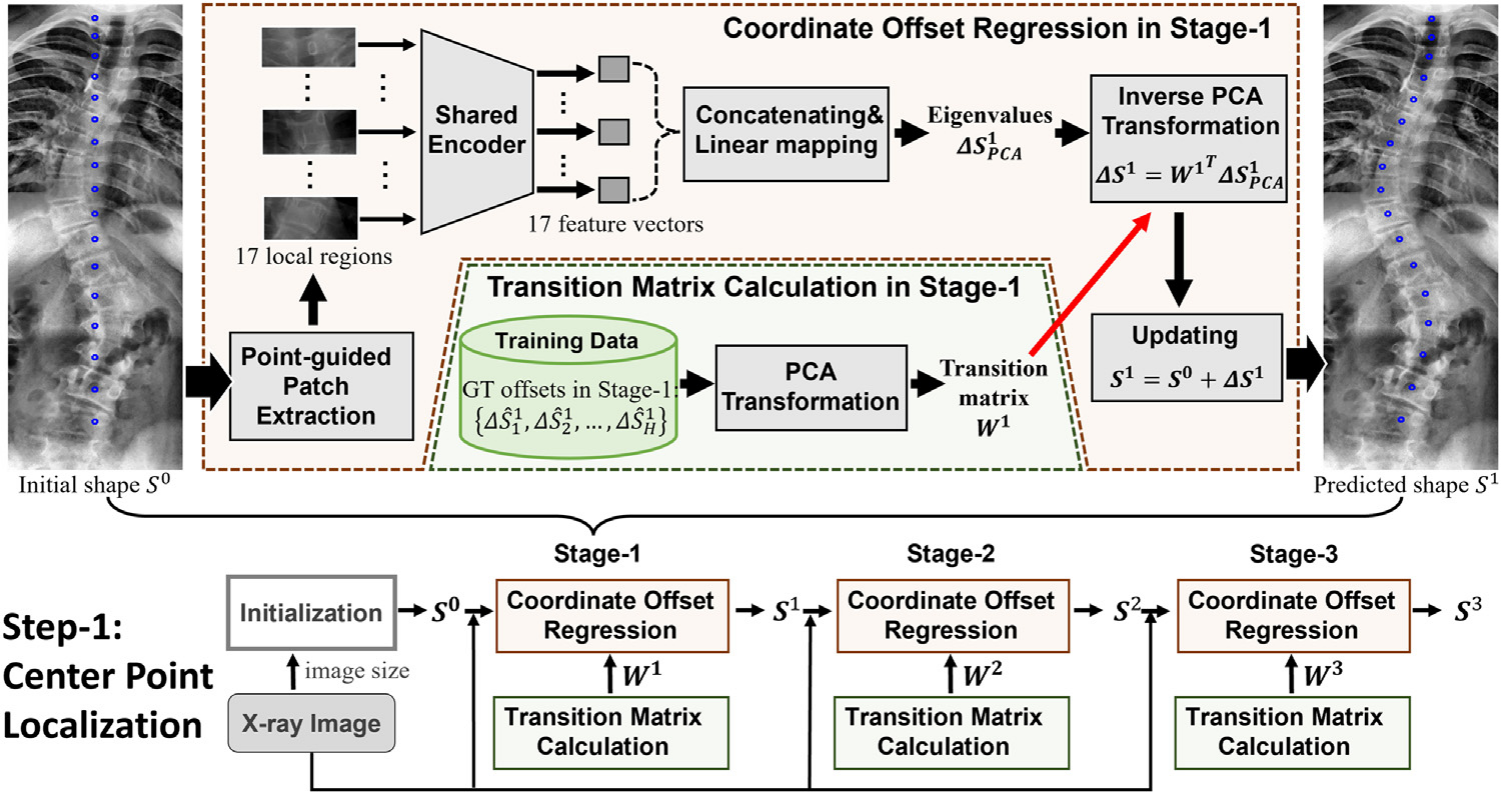
**Overview of the multi-stage cascaded framework in the first step, i.e., center point localization**.

To summarize, our contributions include

(1) We propose a multi-stage regression framework for accurate and robust vertebra landmark localization, in which the former stages identify each vertebra to prevent the latter stages from being disturbed by the neighboring vertebrae.

(2) We regress the PCA-transformed offsets using CNNs in each stage to progressively guide the initially regressed points to the true target positions under a global shape constraint. To the best of our knowledge, it is the first attempt to utilize multi-stage cascaded CNNs for shape-constrained vertebral landmark localization instead of the overused heatmap-based approaches.

(3) Extensive experimental results on the public dataset demonstrate that our method has the advantage of considerably fewer false positives and missing landmarks, and thus achieves superior performance to other state-of-the-arts [2], [3], [5], [9], [10] by decreasing the relative error from 3.2e−3 to 7.2e−4.

## 2 Methodology

As shown in Fig. 1, we address the task of 68 vertebral landmark localization using two sequential steps: seventeen center point localizations for the vertebral column and four corner point localizations for each vertebra. In both steps, we propose a three-stage cascaded framework to gradually update the initial shape (i.e., points) to the ground truth (GT) shape. Each stage contains two major modules, that is, *transition matrix calculation* to mine shape constraints from the training set by PCA and *coordinate offset regression* to update the shape from the previous stage (initial shape if in the first stage).

In the following, we describe the two-step and the two modules.

### 2.1 Step-1: Three-stage framework of center point localization

Our goal in this step is to gradually localize the 17 center points on the entire X-ray image from a set of starting points, that is, $S^{0}=\left(x_{1}^{0},y_{1}^{0},\ldots ,x_{17}^{0},y_{17}^{0}\right)$. To this end, the input image is first proportionally resized to 680-pixel height. The initial shape $S^{0}$ is placed based on the image size, and then gradually updated in three stages. At each stage, (as shown in Fig. 2), the transition matrix calculation first mines the transition matrix from the GT offsets to preserve the shape constraint in the regression. Coordinate offset regression regresses several eigenvalues, which are then converted to shape offset by the transition matrix. An updated shape is obtained by applying the shape offset to the predicted shape from the previous stage (initial shape if in the first stage). The three stages are cascaded to gradually update $S^{0}$ as $S^{n}=S^{n-1}+\Delta S^{n}$, where n=1,2,3, as shown in the bottom of Fig. 2.

#### 2.1.1 Transition matrix calculation

The target of prediction in this step is 34 coordinates of the 17 center points; however, most of them are redundant because the vertebral column roughly fits a curve. In landmark localization, the redundancy in the regression target might greatly increase the chance of losing a shape constraint owing to the uncontrollable regression process of each landmark. PCA [11], [12] is a widely used dimensional reduction approach that can be applied to capture the underlying shape constraint using the first few principal components (i.e., eigenvalues). In addition, the PCA-mined shape constraint can tolerate noise in landmarks because landmark misalignments generally contribute insignificantly to the first few principal components. In this study, we apply PCA to reduce the 34-dimensional regression target to a few eigenvalues, which is detailed as follows.

Given the input shape $S^{n-1}$ in the n-th stage (initial shape $S^{0}$ is in the first stage), the original regression target $\Delta {\hat{S}}^{n}$ is the coordinate offset between $S^{n-1}$ and the GT shape $\hat{S}$, calculated as follows:

(1) $$\Delta {\hat{S}}^{n}=\hat{S}-S^{n-1}\in R^{P},n=1,2,3$$

By collecting $\Delta {\hat{S}}^{n}$ of all H training images, we can obtain a matrix $\Delta {\hat{S}}^{n}$ with a size of P×H, where each column represents a training sample and each row indexes a coordinate, that is, P=34. The covariance matrix C is calculated using Eq. 2:

(2) $$C=\frac{1}{P}\Delta {\hat{S}}^{n}{\left(\Delta {\hat{S}}^{n}\right)}^{T}\in R^{P\times P}$$

Next, we calculate the eigenvalues and the corresponding eigenvectors of the covariance matrix C that satisfy Eq. 3:

(3) $$\lambda_{j}q_{j}=Cq_{j},j=1,\ldots ,P$$

where $\lambda_{j}$ denotes the jth eigenvalue, $\lambda_{1}>\lambda_{2}>\ldots >\lambda_{P}$, $q_{j}$ denotes the corresponding eigenvector. Accordingly, the PCA-derived transition matrix $W^{n}$ can be constructed by selecting the first Q eigenvectors:

(4) $$W^{n}=\left[q_{1}^{T};q_{2}^{T};\ldots ;q_{Q}^{T}\right]\in R^{Q\times P},Q\ll P,n=1,2,3$$

The calculated transition matrix $W^{n}$ in the nth stage can be used to transform the original regression target $\Delta {\hat{S}}^{n}$ into Q eigenvalues according to Eq. 5:

(5) $$\Delta {\hat{S}}_{PCA}^{n}=W^{n}\Delta {\hat{S}}^{n}\in R^{Q},n=1,2,3$$

Comparing Eqs. 5, and 1, we can find that PCA allows us to regress Q eigenvalues instead of P coordinates (Q=8 and P=34 in the first step). Each eigenvalue controls the change in shape in the corresponding eigenvector-defined direction, such as bending, point density, and rotation. Thus, we can avoid dramatic differences between the estimated center points at each stage and those in the training images.

#### 2.1.2 Initialization

After calculating the transition matrix, we perform coordinate offset regression to predict the shape offset for updating. In each stage, the shape offset conditions on the input shape are predicted in the previous stage. Therefore, the shape must be initialized before the *first* stage.

Based on all shapes in the training set, we calculate a normalized averaged shape ${\bar{S}}^{norm}$ for initialization. To this end, each GT shape is normalized according to the size of the corresponding X-ray image:

(6) $${\hat{S}}_{h}^{norm}=\text{normalize}\left({\hat{S}}_{h}\right)={\left(\frac{x_{1}}{\text{Wid}},\frac{y_{1}}{\text{Hei}},\ldots ,\frac{x_{17}}{\text{Wid}},\frac{y_{17}}{\text{Hei}}\right)}_{h},h=1,\ldots ,H$$

where h indexes the GT shape in the training data, H is the total number of training images, ${\hat{S}}_{h}$ is the unnormalized GT shape, $\left(x_{i},y_{i}\right)$ are the coordinates of the ith landmark, Wid and Hei indicate the width and height of the hth X-ray image, respectively.

After normalization, every coordinate is converted to a value ranging from 0 to 1. Subsequently, we calculate the normalized averaged shape as follows:

(7) $${\bar{S}}^{norm}=\frac{1}{H}\sum_{h=1}^{H}{\hat{S}}_{h}^{norm}$$

To initialize in the first stage, we de-normalize ${\bar{S}}^{norm}$ based on the size of the input X-ray image as

(8) $$\begin{array}{cc} S^{0} & =\text{de-normalize}({\bar{S}}^{norm})\\ & =({\bar{x}}_{1}^{norm}\times \text{Wid},{\bar{y}}_{1}^{norm}\times \text{Hei},\ldots ,{\bar{x}}_{17}^{norm}\times \text{Wid},{\bar{y}}_{17}^{norm}\times \text{Hei}) \end{array}$$

where $\left({\bar{x}}_{i}^{norm},{\bar{y}}_{i}^{norm}\right)$ are the normalized coordinates of the ith landmark of ${\bar{S}}^{norm}$. Note that Wid and Hei might vary with different input images; thus, the initial shape changes accordingly.

#### 2.1.3 Coordinate offset regression

Coordinate offset regression sequentially performs feature extraction, regression, and updating based on input shape $S^{n-1}$ as shown in Fig. 2.

**Regression:** At each stage, we extract only features in a local region at every center point from the predicted or initialized shape of the previous stage to capture highly discriminative and noise-resistant features.

We cropped 17 local regions measuring 80×192 guided by the predicted or initialized center points and subsequently resized them to 48×80 for feature extraction using the shared encoder, that is, a truncated MobileNet-V2 [13]. Table 1 lists the architecture details of our truncated MobileNet-V2. The inverted residual block shown in Table 1 first increases the number of channels t times, then convolves the feature maps by a depth-wise convolutional layer with a stride of s (parameter s only works for the first time, and s=1 in the following n−1 repeats), and finally decreases the number of channels to c. Each InvertedResidualBlock operation is repeated n times. More details on the inverted residual block can be found in ref. [13].

**Table 1:** **Architecture of the truncated MobileNet-V2 for feature extraction in both steps.** The size of cropped local regions is 48×80 and 48×48 in the two steps respectively. t: the channel increasing factor in InvertedResidualBlock, c: output channel number, n: the number of the operator repeating, s: stride; the feature maps are spatially compressed using Global average pooling (GAP).

Input size in step-1	Input size in step-2	Operator	t	c	n	s
48×80×1	48×48×1	Conv2d 1×1	-	32	1	2
24×40×32	24×24×32	InvertedResidualBlock	1	16	1	1
24×40×16	24×24×16	InvertedResidualBlock	6	24	2	2
12×20×24	12×12×24	InvertedResidualBlock	6	32	3	2
6×10×32	6×6×32	InvertedResidualBlock	6	64	4	2
3×5×64	3×3×64	InvertedResidualBlock	6	96	3	1
3×5×96	3×3×96	InvertedResidualBlock	6	160	3	2
2×3×160	2×2×160	InvertedResidualBlock	6	320	1	1
2×3×320	2×2×320	Conv2d 1×1	-	64	1	1
2×3×64	2×2×64	GAP	-	-	1	-
Output: 64	Output: 64	-	-	-	-	-

As shown in the first column of Table 1, the shared encoder extracts a 64-dimensional feature vector for each local region, yielding 17 feature vectors in the first step. Owing to both local information of each landmark and shape context of other landmarks matter [14], we concatenate these local feature vectors to form a 1088-d feature vector, followed by a fully connected layer to linearly regress a few eigenvalues $\Delta S_{PCA}^{n}$ as follows:

(9) $$\Delta S_{PCA}^{n}=R\left(S^{n-1};\theta^{n}\right)$$

where $\theta^{n}$ are the parameters of the shared encoder and fully connected layer in the n-th stage, and $R(S^{n-1};\theta^{n})$ is the regression conditioning on the input shape $S^{n-1}$.

**Updating:** The numerical coordinate offsets for the nth stage stage, that is, $\Delta S^{n}=\left(\Delta x_{1}^{n},\Delta y_{1}^{n},\ldots ,\Delta x_{17}^{n},\Delta y_{17}^{n}\right)$, are obtained using inverse PCA transition matrix ${W^{n}}^{T}$ as follows:

(10) $$\Delta S^{n}={W^{n}}^{T}\Delta S_{PCA}^{n}$$

where $W^{n}$ is calculated beforehand as in the transition matrix calculation, and T denotes the transposition operation. The coordinates of the center points from the previous stage $S^{n-1}$ are updated as follows:

(11) $$S^{n}=S^{n-1}+\Delta S^{n}$$

The testing phase is summarized in Algorithm 1. After obtaining $S^{n}$ in the nth stage, we return to the regression process and crop a new set of local regions, guided by $S^{n}$ in the (n+1)-th stage. Because $S^{n}$ is closer to the GT shape than $S^{n-1}$, the extracted features are more discriminative, yielding a more accurate shape offset $\Delta S^{n+1}$ for updating in the subsequent stage. With such stage-by-stage strategy, the shape is updated from $S^{0}$ to $S^{3}$, closer to the GT shape gradually.

**Algorithm 1:**
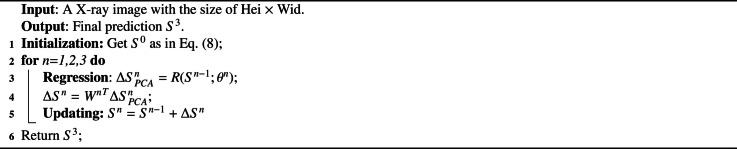
**Pseudo-code for the testing phase of Step-1**.

We optimize $\theta^{n}$ in each stage in the training phase as described below.

**Optimization:** The training phase of the three-stage cascaded CNN for center-point localization is summarized in Algorithm 2. In the nth stage, we first obtain the GT eigenvalues $\Delta {\hat{S}}_{PCA}^{n}$ using Eq. 5 and subsequently predict the eigenvalues $\Delta S_{PCA}^{n}$ based on the previously predicted or initialized shape $S^{n-1}$. The smooth L1 loss function [15] is employed to calculate the loss L between the GT $\Delta {\hat{S}}_{PCA}^{n}$ and the predicted $\Delta S_{PCA}^{n}$ as follows:

(12) $$\begin{array}{c} L=\sum_{i}{\text{Smooth}}_{L_{1}}\left({\Delta S_{PCA}^{n}}_{i}-{\Delta {\hat{S}}_{PCA}^{n}}_{i}\right)\\ {\text{Smooth}}_{L_{1}}\left(x\right)=\left\{ \begin{array}{cc} \frac{0.5x^{2}}{\beta }, & \text{if}\;|x|<\beta \\ |x|-0.5\beta , & \text{otherwise} \end{array}\right. \end{array}$$

where i indexes each eigenvalue and β=0.001 is used to prevent gradient explosions. The parameters $\theta^{n}$ in both the truncated MobileNet-V2 and the fully connected layer are optimized by minimizing L.

**Algorithm 2:**
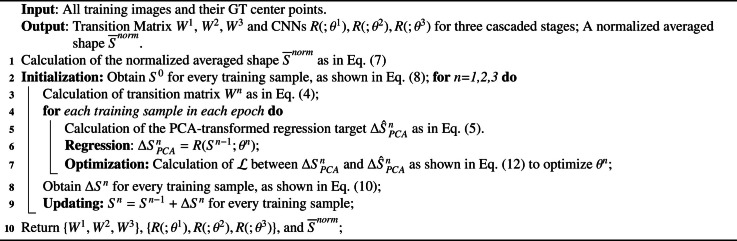
**Pseudo-code for the training phase of Step-1**.

After training, we obtain three transition matrices $W^{1}$, $W^{2}$, $W^{3}$ and the learned parameters $\theta^{1}$, $\theta^{2}$, $\theta^{3}$ for the three cascaded stages, as well as a normalized averaged shape ${\bar{S}}^{norm}$ for initialization.

### 2.2 Step-2: Three-stage framework of corner point localization

**Fig. 3:**
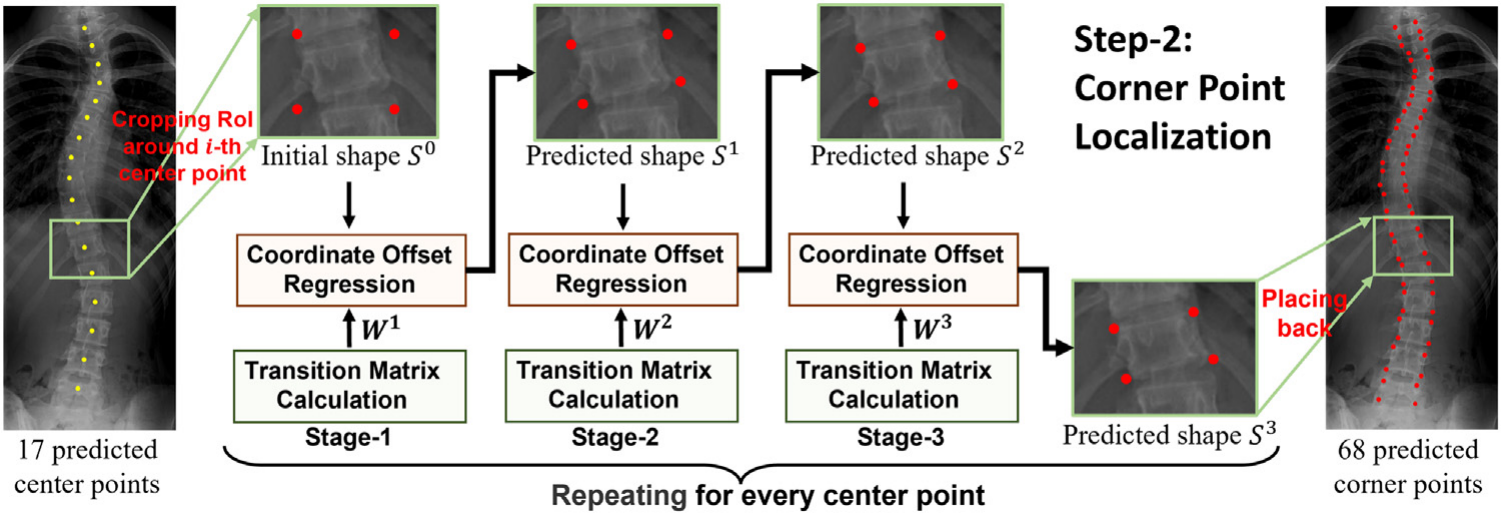
**Overview of the multi-stage cascaded framework in the second step, that is, corner point localization.** 17 RoIs are cropped from the X-ray image at the predicted center points. For each RoI, the localization process is mostly the same as the first step (see Fig. 2) with only two differences, (1) the number of points is 4 instead of 17, (2) initialization is based on the size of RoI instead of the size of X-ray image. By repeating the process for every RoI, 68 corner points can be obtained.

After localizing 17 center points in the first step, we localize four corner points for each vertebra in the second step. Fig. 3 presents an overview of the three-stage cascaded framework for corner-point localization. As observed, most parts are the same as in the first step except that we first extract 17 regions of interest (RoIs) at every predicted center point and repeatedly perform four corner point localization on each RoI instead of the entire X-ray image.

Specifically, an RoI size of 80×96 is extracted with its center location aligned with one predicted center point. We perform initialization, regression, and updating on the extracted RoI as explained. Four initial points are placed based on the size of RoI instead of the entire X-ray image. Four local region sizes of 48×48 for feature extraction in regression were cropped at the predicted or initialized corner points. After the prediction the four corner points for each vertebra were placed on the X-ray image and 68 corner points were obtained, as shown in Fig. 3.

### 2.3 Training details

We used adaptive CLAHE [16] to enhance the X-ray images and applied horizontal flipping to augment the amount of training data twice. We utilize the smooth $L_{1}$ loss with β=0.001 and the Adam optimizer with an initial learning rate of 5e−4 for training. The learning rate was decayed by 0.5 after each epoch. We trained both steps separately in a maximum of five stages and eight epochs for each stage. Our framework was implemented in PyTorch, trained, and tested on a computational platform using a TITAN XP GPU.

## 3 Results

### 3.1 Dataset and evaluation metric

We used the public AASCE dataset^2^, which consists of 609 tight spinal anterior-posterior X-ray images; each image contains 17 vertebrae composed of the thoracic and lumbar spine for spinal shape characterization. Each vertebra was identified by its four corners, resulting in 68 points per spinal image. These landmarks were manually annotated based on visual cues. We utilized two different training-test splits for evaluation: (1) Official Split 2, in which the dataset is split into 481 images for training and the remaining 128 images for validation, as well as an additional 98 challenge-private images with no annotations for testing, (2) Consistent Split [9], in which only the official training set is used, and it is split into 431 for training and 50 for testing, which is consistent with existing state-of-the-art methods [2], [3], [9], [10].

For comparison with the previous research in vertebral landmark localization, we utilized the normalized mean squared error (MSE) as the evaluation metric, which is calculated as follows:

(13) $$f_{mse}=\frac{1}{68}\sum_{i=1}^{68}{\left({\hat{s}}_{i}-s_{i}\right)}^{2}|s_{i}\in S^{norm};{\hat{s}}_{i}\in {\hat{S}}^{norm}$$

where i is the index of each value in $S^{norm}$ and ${\hat{S}}^{norm}$, $S^{norm}$ and ${\hat{S}}^{norm}$ are the normalized estimated and normalized GT coordinates, respectively.

In addition, the AASCE challenge was initialized for estimating three Cobb angles, that is, the proximal thoracic (PT), main thoracic (MT), and thoracolumbar (TL) angles [17], which is a downstream application of vertebral landmark localization. Therefore, we also evaluated our method on a Cobb angle estimation task. The evaluation metric is Symmetric Mean Absolute Percentage (SMAPE), which is calculated as follows:

(14) $$SMAPE=\frac{1}{N}\sum_{j}^{N}\frac{\sum_{i=1}^{3}\left(\left|a_{ji}-b_{ji}\right|\right)}{\sum_{i=1}^{3}\left(a_{ji}+b_{ji}\right)}$$

where i indexes the three Cobb angles, j denotes the jth image, and N is the total number of testing images. a and b are the predicted and ground-truth Cobb angles, respectively.

### 3.2 Comparison with state-of-the-arts for vertebral landmark localization

We evaluated our method on AASCE using Consistent Split, which is in line with previous vertebral landmark localization studies.

**Fig. 4:**
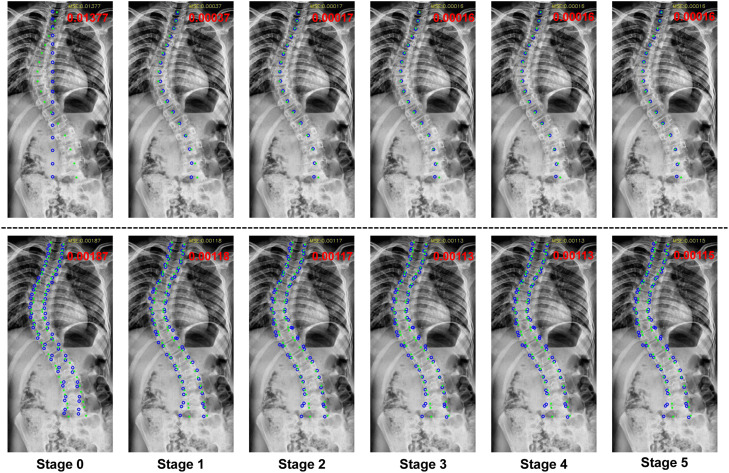
**Our localization performance in different stages.** The top and bottom rows show the localization results of center and corner points, respectively. The green dots and blue circles indicate ground truth and prediction, respectively. The red numbers indicate the MSE.

**Visualization** The visualization results of landmark localization are shown in Fig. 4. It can be observed that for both center point localization and corner point localization, our three-stage cascaded framework can gradually locate almost exactly the same points as the GT corners of the vertebrae stage-by-stage, which effectively recovers both global and detailed structures of the spine. In addition, for both steps, the effect of the first several stages is the most obvious, while the results maintain stability after the third stage. Thus, we consider the prediction of the third stage of each step as the final result of our method.

**Comparison.** We compare our proposed framework with several state-of-the-art methods [2], [3], [5], [9], [10]. Among them, only Yi et al. [5] released their source code; therefore, we used the source code for training and testing based on Consistent Split. The results of $S^{2}\text{VR}$ [2], Conv with dense [10], BoostNet [9] and SLSN [3] were taken from the study of SLSN, and we followed the same standard of data split.

The second column of Table 2 shows the comparison results of different methods. As observed, our method achieves the best performance in terms of MSE (7.2e−4), which is approximately one order of magnitude smaller than [2], [3], [5], [9], and two orders of magnitude smaller than [10]. We believe that the good performance mostly benefits from the multistage cascaded framework and the PCA-mined shape constraint.

**Table 2:** **MSE of landmarks, and the number of parameters and the training time of the two best methods** (i.e., Yi et al. [5] and Ours). The evaluation is performed on 50 images in Consistent split.

Methods	MSE	Number of Parameters	Training Time per Epoch
$S^{2}\text{VR}$[2]	6.0e−3	–	–
Conv with dense [10]	7.1e−2	–	–
BoostNet [9]	4.6e−3	–	–
SLSN [3]	3.9e−3	–	–
Yi *et al.*[5]	3.2e−3	24.21 M	33.69 s
Ours	7.2e−4	11.01 M	38.78 s

These state-of-the-art methods can be divided into two categories: direct regression methods [2], [9], [10] and heatmap-based methods [3], [5]. Compared with direct regression methods, our method uses a multi-stage cascaded framework to progressively predict the center and corner points in two steps, decomposing the difficult task into simpler ones. Compared with heatmap-based methods, our method regresses a few eigenvalues derived by PCA instead of the coordinates, thus better preserving the shape constraint. In comparison, heatmap-based methods essentially segment those pixels close to the landmarks, thus sharing the common issues with the segmentation techniques, that is, scattered false positives incurred by other vertebral-like structures and missing bones induced by the dilution of superimposed tissues.

**Fig. 5:**
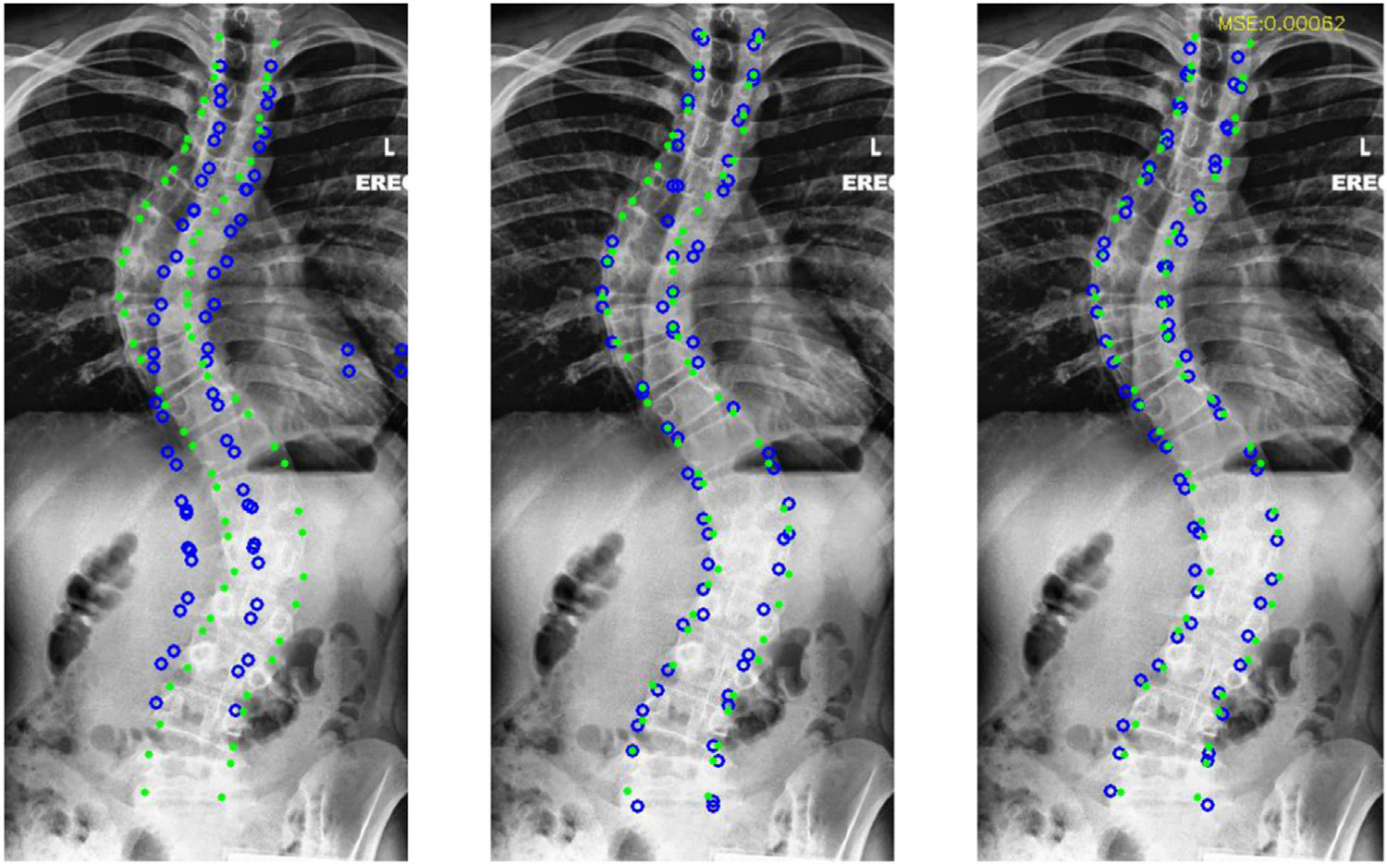
**Example results of direct regression, heatmap-based regression and ours, respectively from left to right.** The green dots and blue circles indicate ground truth and prediction, respectively.

Fig. 5 displays the results achieved by direct regression [10] (implemented for visualization), a heatmap-based approach [5] using U-Net as the backbone [18] and our proposed multi-stage cascaded CNNs. It can be observed that the performance of direct regression is poor, and the heatmap-based approach almost locates the landmarks while suffering from missing localizations induced by the dilution of superimposed tissues, for example, tissues in the lung. Notably, if we manually suppress false positives and exclude missed vertebrae from the evaluation, the error of [5] dramatically decreases to 8.1e−4, which is comparable to ours. This demonstrates that the heatmap-based method suffers from false positives and missing localization in vertebral landmark localization.

**Fig. 6:**
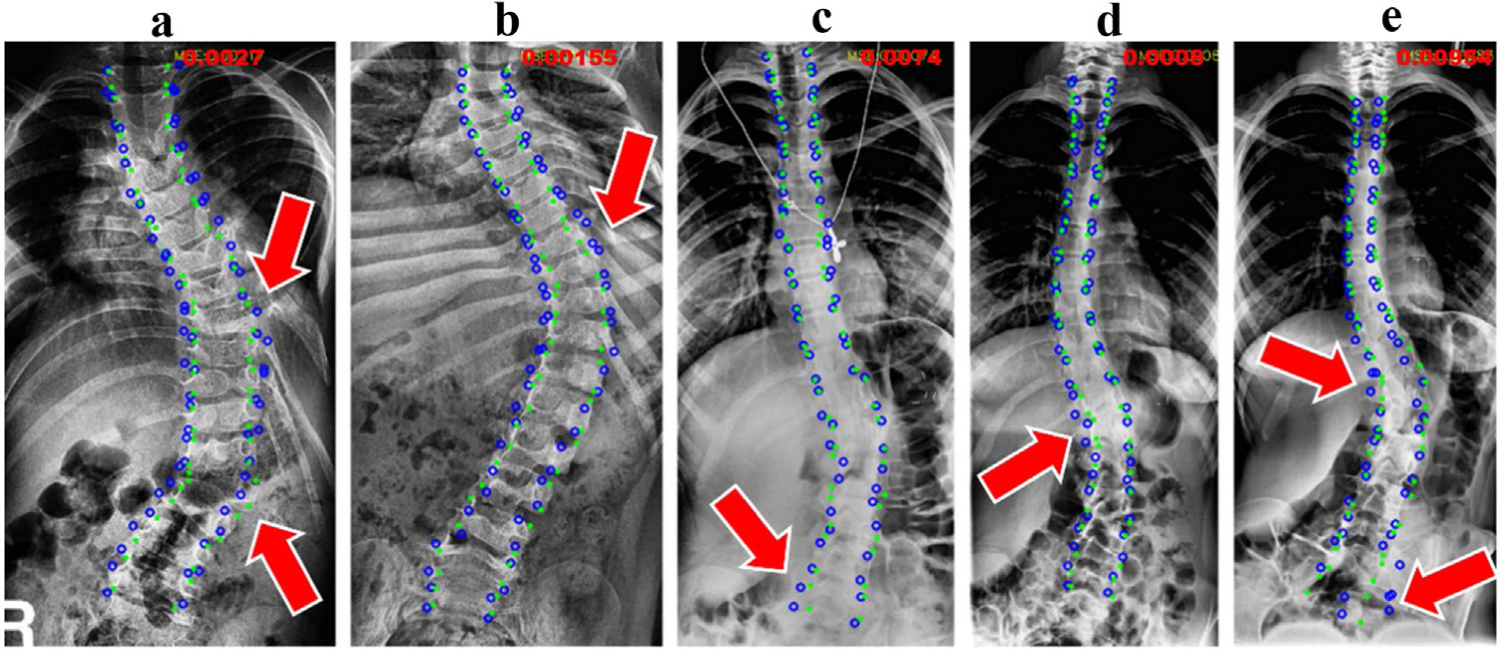
**Five failure cases produced by our method.** A few are due to the confusion between vertebrae and ribs (a-b), and the most are due to the low image contrast (c-e). The green dots and blue circles indicate ground truth and prediction, respectively.

**Failures**. We also demonstrate several failure cases achieved using our method in Fig. 6. It can be observed that most landmarks are successfully localized and the overall shape is accurate, owing to the PCA-mined shape constraint. However, some landmarks remain misaligned with GT, which might be caused by the low image contrast (see Fig. 6c–e) and confusion between the vertebrae and ribs (see Fig. 6a,b).

**Computational cost** We further compare the model size and the training time between the best two methods (i.e., Yi et al. [5] and Ours). Although our method is a multi-stage approach, each stage uses a lightweight CNN, MobileNet-v2, for feature extraction, which was developed for portable devices. Additionally, the last few layers of the original MobileNet-v2 are truncated, further decreasing the number of parameters. Therefore, each stage contains approximately 1.83 M parameters, and six stages (half for center-point localization and half for corner-point localization) have a parameter size of 11.01 M as shown in Table 2.

The training time was recorded by running all data (one epoch) with a batch size of two on the computational platform with a TITAN XP GPU. Results in Table 2 show that, our method outperforms Yi et al. [5] in terms of accuracy for vertebral landmark localization and requires considerably fewer parameters, that is, fewer than that of the others. However, because our model splits the task into two steps, and each step contains three stages, the resulting overhead between every two stages causes slightly more training time than ref. [5] (approximately 5 more seconds per epoch).

### 3.3 Comparison with AASCE challenge participants for Cobb angle estimation

We compared our method with the top-5 participants of the AASCE challenge. The participants evaluated their methods using the estimation error of Cobb angles, while our method predicted the vertebral landmarks. For a fair comparison, we utilize our localized vertebral landmarks to calculate the three Cobb angles. The Cobb angle calculation is realized by an official tool written in MATLAB provided by the challenge organizer, which can automatically convert the input landmarks to three Cobb angles, that is, PT, MT, and TL angles.

We re-trained our method based on the Official Split and evaluated the 98 challenge-private test images, for which the organizer only released the X-ray images for the challenge-private test images but did not provide the ground-truth landmarks or Cobb angles. Therefore, we asked several doctors to manually annotate the vertebral landmarks and utilized the official tool to calculate the Cobb angles as the ground-truth values.

Table 3 shows the results between top-5 participants and the proposed method. Among these participants, three (XMU, iFLYTEK, and XDU)of them first localized 68 vertebral landmarks and then used the official tool to measure the three Cobb angles. The remaining two (Tencent and ErasmusMC) skipped the landmark localization step and directly regressed the Cobb angles based on the entire X-ray image.

**Table 3:** **Comparison results of Cobb angle estimation on the challenge-private test images.** Lower SMAPE value indicates better performance.

Participants	SMAPE
Tencent [19]	21.71%
iFLYTEK [20]	22.17%
XMU [21]	22.18%
ErasmusMC [22]	22.96%
XDU [23]	24.80%
Ours	21.30%

The SMAPE achieved by our method is lower than that of XMU, iFLYTEK and XDU, implying that the landmarks predicted by our method could be more accurate than theirs. Moreover, by obtaining more accurate vertebral landmarks, our performance was the topmost on the Leaderboard,^3^ surpassing the winner Tencent, which directly regressed the Cobb angles without providing the explainable vertebral landmarks.

### 3.4 Ablation study

**Effectiveness of PCA-mined shape constraint** We also validate the effectiveness of PCA-mined shape constraint in the center-point localization step. We developed a non-PCA version of our proposed method, in which the coordinate offset regression module directly regresses the offsets of coordinates instead of the eigenvalues. We calculated the smooth L1 distance between the predicted shape offsets and the GT offsets as the loss to optimize the parameters of the shared encoder and the fully connected layer. Both PCA and non-PCA versions of our method were trained in five stages, and each stage took eight epochs.

**Fig. 7:**
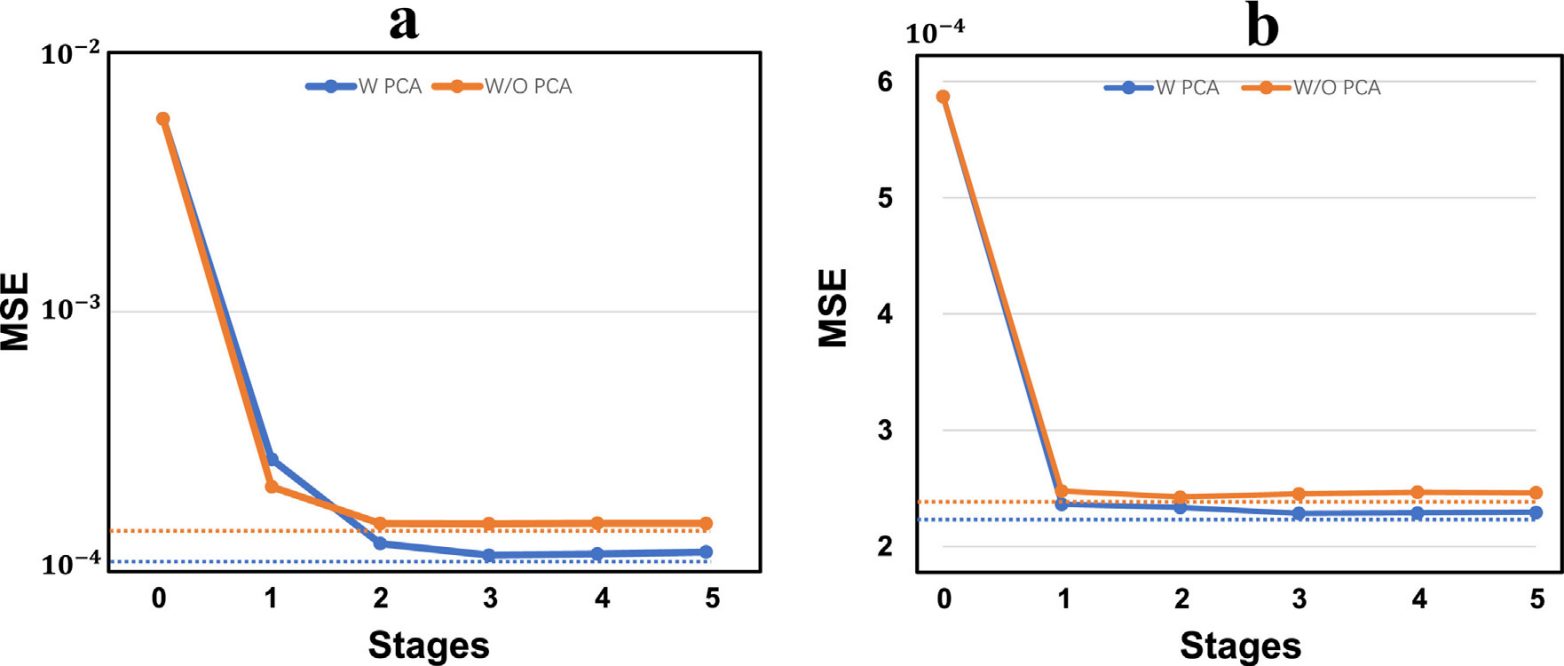
**Illustration of the accuracy improvement via PCA based data pre-processing and best choice of stages.** (a) Center points localization sub-task and (b) corner points localization sub-task based on GT centers, respectively. The blue line and orange line show the MSE with PCA and Without PCA respectively. The horizontal axis represents the stages.

Fig. 7a illustrates the performance at every stage of the two versions of our method for the center-point localization task. The horizontal axis represents the index of the stage and the vertical axis represents the error in the official validation set. At stage-0, the errors are the same, as we utilize the same normalized averaged shape for initialization. For both versions with and without PCA, the error decreases first and achieves the best performance at stage-3, whereas it slightly increases in the later stages because of the overfitting problem. The two dashed lines represent the lowest errors achieved by the two versions at the 3rd stage, respectively, which clearly shows that our proposed PCA-mined shape constraint can boost the performance by a promising margin. In the absence of PCA, the points move independently under no a prior shape constraint, and are thus misaligned with the GT in regions with heavily superimposing tissues or wrongly aligned with the nearby vertebrae.

We subsequently validated the effectiveness of PCA-mined shape constraint in the corner point localization step. Fig. 7b illustrates the comparison results between the two versions with and without PCA, and both were performed on the extracted RoIs at the GT center points to eliminate the influence of step-1. The same conclusions can be drawn from Fig. 7b, that is, both achieve the best performance in stage-3, and the method with PCA surpasses that without PCA, which demonstrates the effectiveness of our proposed PCA-mined shape constraint for landmark localization. Notably, when comparing Fig. 7a,b at stage-0 the initial error of the center points is much higher than that of the corner points because of the relatively higher variance of the spinal shape and fixed structure of the vertebra.

**Sensitivity to initialization.** We further validated the sensitivity of our multistage cascaded framework to initialization. To this end, we disturbed the initial coordinates by adding a random value obtained from a Gaussian distribution $∼N\left(0,\sigma^{2}\right)$ to each of the normalized coordinates of the initial shape. The standard deviation σ controls the distribution severity. By increasing σ, the MSE between the initialization and ground truth increases accordingly; therefore, we could evaluate the sensitivity of our method to different initializations.

**Fig. 8:**
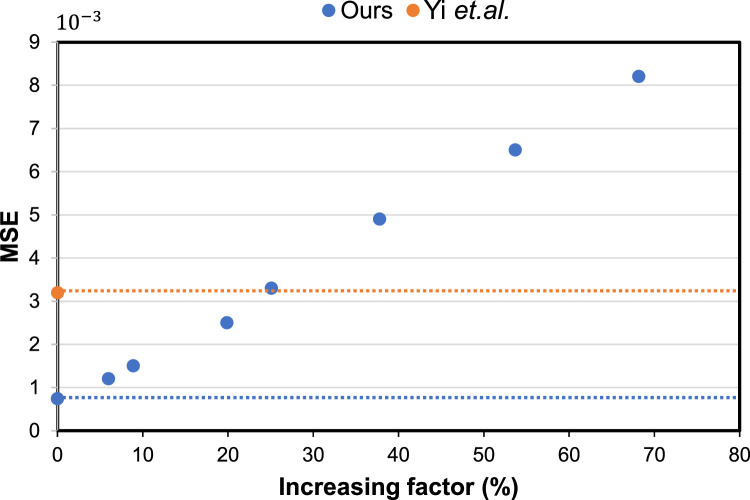
**Validation of the sensitivity to the initialization**.

As shown in Fig. 8, the horizontal axis represents the increasing factor of the initial MSE, and the vertical axis represents the error of the final estimated corner points on the test images of the consistent split. The blue dashed line indicates that the final MSE was 7.2e−4 when the initial coordinates with MSE of 1.4e−2 were not disturbed. As the initial MSE increased, the final MSE error increased linearly, as indicated by the blue dots. It can be observed that our method is relatively robust to initialize and can maintain a superior performance (MSE <3.2e−3) to the comparison methods (e.g., the orange dashed line indicates the result of ref. [5]), given distributions less than 24.6%.

## 4 Discussion

Vertebral landmark localization on X-ray images for patients with scoliosis is a crucial step in providing clinically valuable biomarkers for various downstream applications, such as the Cobb angle estimation and vertebra identification; however, this approach is extremely challenging because of the low contrast induced by superimposing tissues and incorrect misalignment with nearby vertebrae. Existing solutions mainly use two approaches: a heatmap-based approach and a regression-based approach. The heatmap-based approach has been widely used for landmark localization in the field of computer vision and has been demonstrated to handle objects with large poses, such as facial landmarks or human poses. However, this approach is overused because the vertebral column roughly fits a curve with only a slight variation under a clear prior of the shape constraint. However, the side effects of these heatmap-based approaches include their application in vertebral landmark localization and often suffer from false positives and missing bones. The regression-based approach can guarantee a one-to-one match between the predicted and the GT landmarks, but it often fails to regress 68 vertebral landmarks at once, especially for these homogeneous vertebrae.

In this study, we address the difficult task of vertebral landmark localization in two sequential steps: i.e. first, localizing 17 center points of the vertebrae and second, localizing 68 corner points around the predicted center points in the first step. Thus, the difficulty decreases in both steps, which can be easily addressed by our model. In the second step, our model focuses on every single vertebra, preventing the localization process from being disturbed by other nearby vertebrae. Furthermore, in each step, we design a multi-stage cascaded network with a PCA-based shape constraint to further decrease the difficulty, which is inspired by the philosophy of a boosting algorithm called the gradient boosting decision tree (GBDT) [24]. GBDT solves a difficult problem using multiple weak learners in multiple stages, and each learner learns to fix the unsolved problem remaining from the last learner. Similarly, we trained three CNNs in three stages, and each CNN is only responsible for predicting the offset between the GT and the prediction from the previous stage. We employed the lightweight MobileNet-V2 as the weak learner; therefore, our method has only a few parameters but achieves a promising performance.

Extensive and comprehensive experiments demonstrate the superior performance of our method in the task of vertebral landmark localization compared with the existing state-of-the-art methods, as well as the task of downstream Cobb angle estimation compared with the AASCE challenge participants^3^. Furthermore, we demonstrate the effectiveness of our proposed PCA-mined shape constraint for vertebral landmark localization, which allows us to gradually update the initial shape to the GT shape in several eigenvalue-defined directions, such as bending and scaling, instead of moving landmarks independently and uncontrollably.

The study of failure cases clearly shows that although there are a few misaligned landmarks, the rough shape of the vertebral column is well preserved. These misaligned landmarks are mainly caused by the confusion of ribs and highly superimposed tissues, verifying the difficulty of vertebral landmark localization on X-ray images in scoliosis patients.

In the future, we will introduce an end-to-end training strategy into this work, thus making the two steps and cascaded CNNs mutually guide each other, expecting to further boost the model performance. In addition, we will extend our approach to other landmark localization tasks, particularly when there are high correlations between the landmarks.

## 5 Conclusion

In summary, this study tackles the complex task of vertebral landmark localization on X-ray images for scoliosis patients by introducing a two-step method: first identifying the vertebral centers and then locating corner points around these centers. By breaking down the challenge into these sequential steps and applying a multi-stage cascaded network with a PCA-based shape constraint, the model addresses both local misalignments and false positives commonly encountered in existing methods. Using lightweight MobileNet-V2 networks as the model's “weak learners” allows efficient performance with fewer parameters. The results demonstrate the model's effectiveness for both landmark localization and downstream tasks like Cobb angle estimation.

## Declaration of competing interest

The authors declare that they have no conflicts of interest in this work.

## Footnotes

2 https://aasce19.github.io/.

3 https://aasce19.grand-challenge.org/evaluation/challenge/leaderboard/.
